# Editorial: Oxidative stress and inflammation in cardiometabolic disorders

**DOI:** 10.3389/fendo.2024.1397836

**Published:** 2024-03-22

**Authors:** Aleksandra Klisic, Dimitrios Patoulias, Esma R. Isenovic

**Affiliations:** ^1^ Department for Biochemistry, University of Montenegro-Faculty of Medicine, Podgorica, Montenegro; ^2^ Center for Laboratory Diagnostics, Primary Health Care Center, Podgorica, Montenegro; ^3^ Outpatient Department of Cardiometabolic Medicine, Second Department of Cardiology, Aristotle University of Thessaloniki, General Hospital “Hippokration”, Thessaloniki, Greece; ^4^ Department of Radiobiology and Molecular Genetics, VINČA Institute of Nuclear Sciences - National Institute of the Republic of Serbia, University of Belgrade, Belgrade, Serbia

**Keywords:** obesity, diabetes, oxidative stress, inflammation, cardiometabolic risk

Obesity and obesity-related illnesses, such as metabolic syndrome (MetS), type 2 diabetes mellitus (T2D), and non-alcoholic fatty liver disease (NAFLD), are becoming increasingly prominent around the world (1, 2). These disorders are linked and share common pathophysiological characteristics, including dyslipidemia, insulin resistance, systemic low-grade inflammation, and oxidative stress (3, 4).

The imbalance between the antioxidant defence system and reactive oxygen and nitrogen species affects the function and structure of proteins, lipids, and nucleic acids. Furthermore, the deregulation of adipose tissue-secreted cytokines and adipokines influences insulin signaling pathways, resulting in a vicious cycle of inflammation/oxidative stress and cardiometabolic diseases (5, 6).

This Research Topic investigated current trends and breakthroughs in cardiometabolic illnesses, focusing on identifying novel diagnostic/prognostic/therapeutic biomarkers for these conditions.

Teaney and Cyr provided an intriguing narrative review on the potential role of Forkhead box O1 (FoxO1) protein in the pathogenesis of T2D, its expression in various tissues and organs commonly affected by T2D, and, most importantly, its potential role as a therapeutic target in the most impactful metabolic disease to date. However, no substantial evidence demonstrates that targeting FoxO1 has a therapeutic advantage for T2D patients. As a result, additional research is needed to answer this highly intriguing research hypothesis.

In a nationwide prospective study, Qian et al. examined the utility of a simple and cost-effective algorithm, Mets-IR, which integrates body mass index (BMI) and common laboratory indicators such as fasting blood glucose, triglycerides, and high-density lipoprotein cholesterol (HDL-C). They confirmed its link to an increased risk of stroke and cardiovascular disease, demonstrating that low-density lipoprotein cholesterol (LDL-C) mediates these relationships. This implies that improving insulin sensitivity and lipid regulation could be necessary preventive measures for cardiovascular diseases/events.

Bariatric surgery and medication treatment are additional alternatives for treating obesity-related diseases if weight-loss programs and physical exercise fail to provide desired outcomes as the first line of treatment (7, 8).

Butler et al. conducted a cross-sectional analysis at a 12-year follow-up of obese individuals with and without a history of Roux-en-Y bariatric surgery. The results indicated a statistically significant improvement in cardiometabolic parameters of interest for the bariatric surgery group. The hepatokine, adipokine, and myokine markers of body homeostasis, which were not fully normalized, showed that they were still fat despite that improvement, as per the pertinent studies carried out by the study group.

Kwon et al. showed that treatment with orlistat plus phentermine, as opposed to placebo plus phentermine, was associated with a significant improvement in oxysterol metabolism, as evidenced by the significant decrease in serum levels of sterols, free cholesterol, sitosterol, 7α-hydroxycholesterol (7α-OHC), and 7β-OHC at 12 weeks in the 12-week randomized, double-blind, controlled trial that enrolled obese individuals. These results are relevant because they highlight the critical role oxysterol plays in the onset and progression of atherosclerosis and because people with preexisting obesity bear a disproportionately high risk of cardiovascular disease.

In a mouse model of NAFLD, semaglutide, a glucagon-like peptide-1 agonist, improved liver outcomes, pro-inflammatory biomarkers, body weight, and metabolic parameters. Niu et al. revealed that semaglutide inhibited the upregulation of multiple pro-inflammatory factors and reduced (but did not normalize) hepatic inflammation.

This Research Topic summarizes the current methods for managing cardiometabolic risk and disease. It also offers some treatment options for lowering obesity and cardiometabolic risk and some straightforward, affordable, and easily accessible diagnostics and prognostic indices that include laboratory biomarkers and anthropometric indices. More studies are required to confirm these findings and enhance the prognostic, therapeutic, and diagnostic approaches for these conditions.

## Author contributions

AK: Writing – original draft. DP: Writing – review & editing. EI: Writing – review & editing.
